# Commentary: An ounce of prevention

**DOI:** 10.1016/j.xjtc.2021.01.015

**Published:** 2021-01-20

**Authors:** Lawrence M. Wei, Vinay Badhwar

**Affiliations:** Department of Cardiovascular and Thoracic Surgery, West Virginia University, Morgantown, WVa

**Figure:**
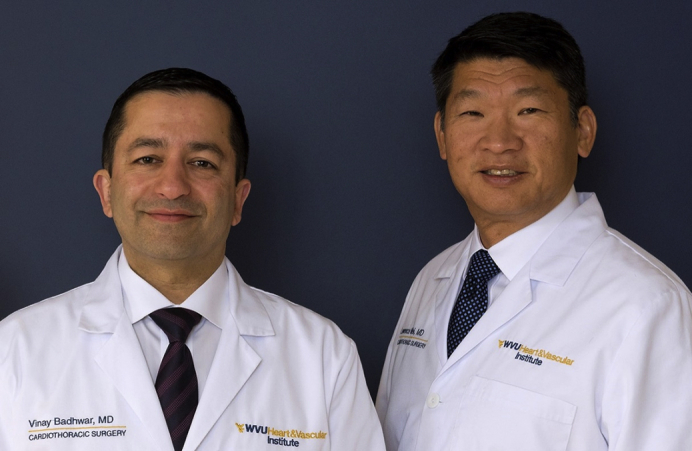
Vinay Badhwar, MD (*left*), and Lawrence M. Wei, MD (*right*)

**Central Message**

Preventing the need for reoperation following transcatheter aortic valve replacement begins with careful patient selection and weighing other durable low-risk surgical alternatives.

See Article page 54.

In this issue of *JTCVS Techniques*, Burke and colleagues^1^ present a timely review of surgery following transcatheter aortic valve replacement (TAVR). As TAVR becomes more prevalent and its application attempts to expand to younger and lower-risk patients, cardiac surgeons must be equipped to address acute and late failures that require surgical intervention. The innovation of TAVR has caused a new surgical pathology, and our colleagues^1^ have provided a pathway to prepare for what is come.

Acute “bailout” surgery for intraprocedural complications of TAVR has an incidence of <4% but significant mortality and morbidity.^2^^,^^3^ Cardiac perforation, tamponade, device migration, and annular rupture are the most common reasons for intervention, with aortic dissection and coronary occlusion requiring surgery less common.^4^ Although perforation is a risk of any transcatheter procedure, device migration is potentially preventable, as it is clearly associated with certain factors, including aortic insufficiency, large annuli, minimal annular calcification, non-coaxial horizontal alignment, and bicuspid valve pathology, that all tend to favor surgical aortic valve replacement (SAVR).^5^^,^^6^ Avoidance of TAVR for such patients, particularly if low to intermediate risk, would mitigate bailout and may increase a program's quality outcomes. As we soon enter a period of TAVR public reporting, perhaps teams will be more careful in selecting TAVR over SAVR.

Current support for TAVR in low-risk patients is based on the PARTNER 3 trial (SAPIEN S3, Edwards Lifesciences, Irvine, Calif) and Evolut Low Risk trial (CoreValve, Medtronic, Minneapolis, Minn).^7^^,^^8^ The PARTNER 3 trial included rehospitalization, stroke, and all-cause mortality in the composite end point and the Evolut Low Risk trial employed a composite end point of stroke and all-cause mortality with noninferiority to SAVR. Although both trials met their designed primary end points, isolated mortality of TAVR and SAVR were similar. Importantly, both trials excluded many of the patients who now are being referred for TAVR (younger, bicuspid pathology, subvalvular calcium, anatomic size exclusions). With more young and low-risk patients being referred for TAVR, it is incumbent upon the multidisciplinary heart team to consider carefully and objectively whether TAVR is reasonable when low-risk, minimally invasive SAVR is available.^6^ Preventing a complication by avoiding an inappropriate procedure is preferable to managing it with a high-risk emergent intervention.

Burke and colleagues^1^ appropriately emphasize the importance of formal discussion by the multidisciplinary heart team with the patient and family of the risks of complications as well as candidacy and prognosis for salvage interventions. Candidacy for bailout and detailed plans for the approach should be reviewed by the procedural team as part of the preprocedure briefing. Clearly defining which patients are not candidates for bailout procedures will help to prevent futile salvage operations. The authors^1^ nicely outline the technical challenges, such as adherence of the prosthesis to the native leaflets and ingrowth of the prosthetic material into the subannular and supra-annular tissue, especially with a self-expanding prosthesis. Aortic root replacement, with its attendant complexity and greater risk, frequently is required. These are issues that do not occur with a conventional redo-SAVR operation.

Paravalvular leak (PVL), prosthetic valve endocarditis, structural and hemodynamic valve deterioration, thrombosis, and late migration are the usual indications for late surgical intervention following TAVR. Although the incidence and severity of PVLs has decreased with newer generations of TAVR prostheses, they still occur with greater frequency than with SAVR and not all are repairable with catheter-based closure devices.^7^, ^8^, ^9^, ^10^

**Figure 1:**
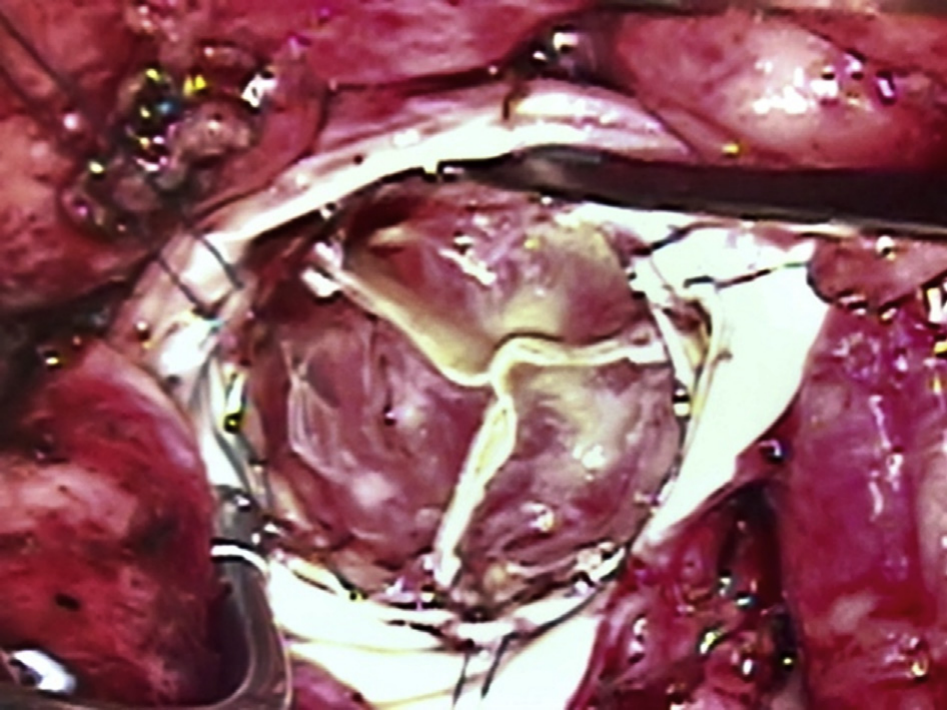
Reoperation for prematurely thrombosed and stenotic transcatheter aortic valve replacement in a previously low-risk 71-year-old man.

The long-term durability of TAVR prostheses, still an unknown, is unlikely to exceed nor match that of SAVR. Five-year data on intermediate-risk patients show rates of PVL, need for reintervention, and longitudinal results that may begin to favor SAVR over TAVR.^9^ TAVR, the revolutionary technology originally intended as therapy for patients too old or frail to be surgical candidates and for whom durability was irrelevant, now is being applied to younger patients who are at low risk for surgical procedures and for whom durability is the most important consideration. Although the index procedural risk may be similar for TAVR and SAVR, the long-term outlook must factor in the shorter durability of the TAVR prosthesis and the greater risk of repeat procedures. Some patients with degenerated TAVR prostheses will be able to undergo valve-in-valve TAVR, a procedure with mixed results and even less durability data than primary TAVR,^10^^,^^11^ but most will require reoperative SAVR and be subject to the increased reoperative surgical risks (Figure 1). Performing low-risk SAVR as the index procedure in a younger patient will reduce the likelihood of greater risk reoperation later in life.

Newer approaches to SAVR, including robotically assisted minimally invasive AVR, may offer reduced morbidity, shorter hospitalization, and more rapid recovery while providing the proven track record of conventional SAVR prostheses and elimination of the short- and long-term risks of reoperation for failed TAVR.^6^ For some young low-risk patients, it is unconscionable to sacrifice the long-term benefits of SAVR for the short-term convenience of TAVR. Burke and colleagues^1^ are to be commended for providing this frank commentary on the risks of surgery following TAVR. Their findings support the adage that “an ounce of prevention” with careful selection of SAVR over TAVR for those with predictable features, “is worth a pound of cure” to avoid early reoperation.
